# Accuracy of Colon Capsule Endoscopy in Detecting Colorectal Polyps in Individuals with Familial Colorectal Cancer: Could We Avoid Colonoscopies?

**DOI:** 10.1155/2017/1507914

**Published:** 2017-02-07

**Authors:** Cristina Alvarez-Urturi, Gloria Fernández-Esparrach, Inés Ana Ibáñez, Cristina Rodríguez De Miguel, Josep Maria Dedeu, Xavier Bessa, Henry Córdova, Maria Pellisé, Francesc Balaguer, Angels Ginés, Luis Barranco, Isis K. Araujo, Montserrat Andreu, Josep Llach, Antoni Castells, Begoña González-Suarez

**Affiliations:** ^1^Department of Gastroenterology, Hospital del Mar, UAB, Parc de Salut Mar, Barcelona, Catalonia, Spain; ^2^Gastroenterology Department, Endoscopy Unit, ICMDiM, Hospital Clinic, CIBEREHD, IDIBAPS, University of Barcelona, Catalonia, Spain

## Abstract

*Background*. Individuals with a family history of colorectal cancer (CRC) have an increased risk of CRC. We evaluated the diagnostic yield of CCE in the detection of lesions and also two different colon preparations.* Methods*. A prospective multicenter study was designed to assess CCE diagnostic yield in a cohort of asymptomatic individuals with a family history of CRC. CCE and colonoscopy were performed on the same day by 2 endoscopists who were blinded to the results of the other procedure.* Results*. Fifty-three participants were enrolled. The sensitivity, specificity, PPV, and NPV of CCE for detecting advanced adenomas were 100%, 98%, 67%, and 100%. Sensitivity, specificity, PPV, and NPV of CCE for the diagnosis of individuals with polyps were 87%, 97%, 93%, and 88%, respectively. CCE identify 100% of individuals with significant or advanced lesions. Overall cleanliness was adequate by 60.7% of them. The PEG-ascorbic boost seems to improve colon cleanliness, with similar colonic transit time.* Conclusion*. CCE is a promising tool, but it has to be considered as an alternative technique in this population in order to reduce the number of colonoscopies performed. More studies are needed to understand appropriate screening follow-up intervals and optimize the bowel preparation regimen.

## 1. Introduction

Most colorectal cancer (CRC) cases are sporadic but familial clustering is found in 25%. It is well known that the risk of developing CRC is 2 to 4-fold higher in individuals with a family history of CRC than in the general population, depending on the number of cases of CRC in the family and how closely the individuals are related [1]. A recent Chinese study [2] has also shown that the prevalence of advanced neoplasm (defined as CRC or adenoma of at least 10 mm with high-grade dysplasia or villous component in histology) is higher in the siblings of patients with CRC than in controls (7.5%* versus* 2.9%, odds ratio [OR] 3.07, *P* < 0.05). Thus, in this group of high-risk individuals, it is recommended that screening begin at an earlier age and be conducted with increased frequency [2]. For instance, screening in individuals with a first-degree relative diagnosed with CRC before the age of 60 years or in persons with 2 or more cases in the family diagnosed at any age should begin at the age of 40 years or 10 years before the age at diagnosis of the relative. The current recommendation for this group is to perform CRC screening with colonoscopy every 5 years [2–4].

However, colonoscopy, which is considered the gold standard, has some limitations that may affect compliance. One of them is perception of the test, which varies by country, ethnicity, and socioeconomic status. Recent published data from a multicenter study showed that colonoscopy participation rate in a population screening program was only 24.6% [5]. The risk of potential complications is another limitation. Rate of perforation after a diagnostic colonoscopy is very low (1/1000 patients) but exists [6].

Another important drawback of colonoscopy is the risk of missed lesions, which, according to a recent study, occurs in 2% to 26% cases [7]. Studies also suggest that colonoscopy may not be as sensitive in the right colon as it is in the left colon [8, 9]. Then other alternatives have been explored [10].

Colon capsule endoscopy (CCE) allows visualization of the colon mucosa without the need for sedation and insufflation. Since its introduction in 2006, this procedure has demonstrated to be safe and well-tolerated [10–17]. CCE has emerged as a potential cost-effective alternative to colonoscopy because it may improve adherence to CRC screening programs, although this has not been demonstrated yet [18]. Therefore, the objective of this study was to assess the diagnostic yield of CCE in the detection of significant colonic lesions (polyps and cancer) in individuals with a family history of CRC and to compare the efficacy of two different colon preparations.

## 2. Patients and Methods

### 2.1. Study Group

Individuals with CRC familial history were recruited from colonoscopy screening schedules. The protocol was approved by the Ethics Committee of Hospital Clinic and participants gave informed consent.

Participants were prospectively enrolled from January 2009 to January 2011 in 2 tertiary hospitals in Barcelona. CCE and colonoscopy were performed on the same day by 2 different endoscopists who were blinded to the results of the other examination.

Inclusion criteria included asymptomatic individuals with a first-degree relative diagnosed with CRC before the age of 60 years or with 2 or more relatives diagnosed with CRC at any age. Exclusion criteria were previous history of colonic lesions (polyps and/or neoplasia, inflammatory bowel disease, polyposis syndromes, or Lynch syndrome), severe heart failure or renal failure, dysphagia, suspicion of intestinal obstruction, or pregnancy.

### 2.2. CCE

CCE was performed using the first-generation Pillcam® Colon capsule (Given Imaging, Israel) which measures 31 × 11 mm and acquires images at a rate of 4 frames per second. All capsule videos were read by 2 physicians with expertise in this technique. A dissolvable capsule (Agile® Patency Capsule, Given Imaging) was administered prior to the procedure in case of major previous intestinal surgery, chronic use of nonsteroidal anti-inflammatory drugs, or abdominal pain to rule out stenosis. Exploration was considered complete when hemorrhoids plexus were seen or capsule was excreted before colonoscopy.

### 2.3. Colon Preparation

Preparation started the day before with a diet consisting of liquids and carbohydrates (plain noodles cooked in salt water) at lunch time in order to increase motility. At night and the next morning, participants ingested a low-volume preparation containing polyethylene glycol (PEG) plus ascorbic acid in split dose (Moviprep) (Table 1). As boosters they took PEG + ascorbic (group 1) or sodium phosphate (group 2) in order to compare colon cleanliness and transit time.

Similarly to previous studies, a 4-point scale grading system for the evaluation of colon preparation was used [11]. Colon cleanliness was scored as excellent (no more than small pieces of adherent feces), good (small amount of feces or dark fluid but not enough to interfere with the examination), fair (enough feces or dark fluid present to preclude a completely reliable examination), or poor (large amount of faecal residue) in 5 segments (cecum, ascending, transverse, descending-sigmoid, and rectum). An overall colon cleansing grade was also evaluated by using the same grading system. It was considered an adequate preparation when excellent or good and inadequate if fair or poor.

### 2.4. Colonoscopy (Gold Standard)

Colonoscopy was performed in the afternoon, between 8 and 10 hours after capsule ingestion, under sedation (midazolam or propofol at the physician's discretion). Cleansing was graded upon colon withdrawal for each segment after completion of washing with the Boston Bowel Preparation [19]. Polyps detected during colonoscopy were removed and sent for histological assessment. If the capsule was seen during colonoscopy, it was retrieved.

Significant polyps were considered as those with size ≥6 mm. Advanced adenomas were considered those ≥10 mm, with villous histology or high-grade dysplasia. When a polyp was identified by CCE but not in colonoscopy, the patient was rescheduled for a second colonoscopy. False positives were defined as lesions detected only by CCE, after 2 consecutive colonoscopies.

### 2.5. Satisfaction Level

A patient satisfaction questionnaire consisting of 3 questions was done telephonically one month later. Patients were asked to evaluate their degree of satisfaction with the CCE and colonoscopy procedures using a 5-point subjective scale (from poor to excellent).

### 2.6. Statistical Analysis

Sample size was calculated assuming that CCE would detect the same number of lesions than colonoscopy, giving a total of 51 patients, with a significance level of 5%, statistical power of 80% assuming 10% of dropout rate.

Performance characteristics and 95% CI were calculated for any-sized polyps and for polyps 6 mm or larger. Descriptive statistics for continuous variables are expressed as mean and standard error and categorical variables as percentage. A two-sided Student's *t*-test was used to compare continuous variables, and the *χ*^2^ test was applied to compare categorical variables. Statistical analysis was performed with SPSS software, version 19.0.

## 3. Results

A total of 53 participants were eligible for inclusion but two were excluded because of technical CCE failure and incomplete colonoscopy, respectively. Therefore, 51 participants were included and data analyzed: 27 in group 1 (boosters with ascorbic) and 24 in group 2 (boosters with sodium phosphate). Eighteen individuals (35.2%) had a total of 51 polyps. Demographic characteristics of the participants are shown in Table 2. Characteristics of the lesions detected by colonoscopy and CCE are shown in Table 3.

Capsule Agile Patency was administered to 4 patients confirming intestinal permeability and CCE was ingested without problem.

### 3.1. Per Individuals Analysis

CCE detected polyps in 15 individuals (29.4%). In four patients in whom CCE was normal and colonoscopy detected lesions, all of them were nonsignificant lesions. One of them had poor preparation and 5 polyps were missed and the other 3 subjects had good preparation and 4 polyps were missed. Histological analysis of these lesions was tubular adenoma (*n* = 3) and hyperplastic polyps (*n* = 6).

Sensitivity, specificity, PPV, and NPV of CCE for the detection of individuals with polyps of any size were 87%, 97%, 93%, and 88% respectively. Remarkably, when we analyzed only subjects with significant or advanced lesions, CCE identified all of them.

### 3.2. Per Polyps Analysis

CCE detected 38 polyps and colonoscopy 51 polyps. Four polyps were seen by CCE and not in the first colonoscopy. However, the second colonoscopy found a 4 mm polyp that was removed and the other 3 were considered false positives. Therefore, 17 polyps in 8 individuals were missed by CCE. All of them were nonsignificant lesions. 14 of these polyps were hyperplastic and 3 were tubular adenoma. None of the missed polyps showed a villous component or high-grade dysplasia.

The diagnostic yield, sensitivity, specificity, PPV, and NPV of CCE are shown in Table 4. No cases of CRC were diagnosed.

Potential factors associated with missed polyps including complete examination with CCE, CCE cleanliness, type of booster, or location of polyps were included in an univariate analysis. Only location was a significant risk* factor* from univariate and was included in a multivariate analysis. Finally, the only independent predictor for missed polyps in our study was location in the left colon, rectum essentially (OR 0.4; *p* < 0.001).

### 3.3. Excretion Rate and Colon Cleanliness

The mean transit time from mouth to the anus was 251.0 ± 17.9 min.

CCE was excreted within 10 hours of ingestion in 84.3% of the participants. Patients of group one had a shorter colonic transit time (128,5 ± 95.6 versus 166.8 ± 121 minutes, pNS). CCE examination was complete in 44 individuals (87%) and incomplete in 7 cases (13%; 6 in group 1 and only 1 in group 2); in these cases capsule was retrieved during colonoscopy in the rectosigmoid area in 5 participants and in the cecum in 2 of them.

**Figure 1:**
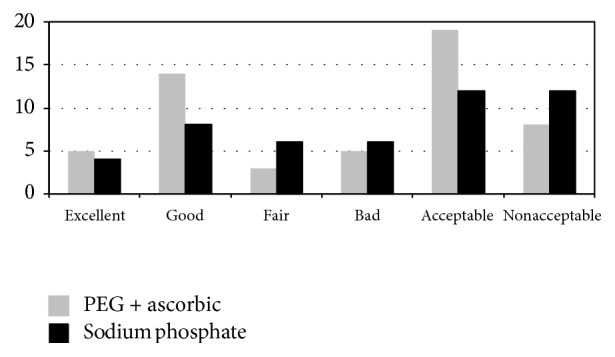
Number of patients with acceptable preparation in capsule endoscopy with different boosters (*p* = 0.1).

Overall cleanliness was excellent or good in 60.7% of CCE examinations (70.3% in group 1 and 50% in group 2, *p* = 0.1) (Figure 1). Colon cleanliness was graded better in the group of PEG + ascorbic boost compared to the cleanliness in the sodium phosphate boost group but this difference was not statistically achieved.

### 3.4. Safety and Tolerability

**Figure 2:**
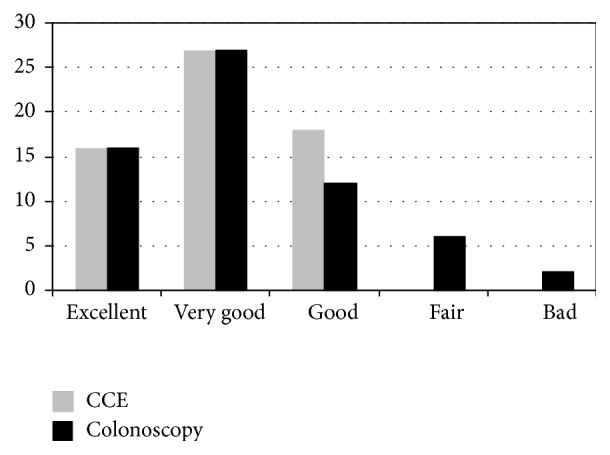
Patients satisfaction level (%) with colon capsule and colonoscopy.

There were no adverse effects related to CCE, colonoscopy or bowel preparation. Only 31 out of 51 subjects answered the satisfaction questionnaire. CCE was evaluated as excellent, very good, or good in 100% of participants, while 16% of them evaluated colonoscopy as fair-bad. Overall, 70.9% of participants submitted to the questionnaire preferred CCE, while 29% preferred colonoscopy (Figure 2).

## 4. Discussion

In this prospective, multicenter study specifically designed to assess CCE diagnostic yield in asymptomatic subjects with a family history of CRC, we can conclude that CCE is an accurate, feasible, well-tolerated, and safe alternative for this CRC high-risk population. CCE was able to identify all the individuals with significant polyps, using a low-volume preparation regimen with very good tolerance and acceptable cleanliness level. Most important target is to identify patients to be submitted to colonoscopy (with any significant polyp), with a noninvasive test. This strategy could be accepted for a higher number of subjects.

Sacher-Huvelin et al. analyzed a cohort of 545 patients at average risk (*n* = 163) or increased risk of CRC (*n* = 376) and described a low overall sensitivity for polyp detection, probably due to poor colon cleanliness [17]. Gay et al. also analyzed 128 individuals with any indication for colonoscopy (76% with excellent or good preparation) and showed sensitivity for CCE of 87.5% [16]. Our results are in line with more recent studies that have shown that CCE is as effective as colonoscopy for detecting significant lesions [20–23].

The role of colon capsule endoscopy is still unclear and it seems a contradiction to use an alternative technique in a high-risk population because colonoscopy is our gold standard. However, most of these colonoscopies, about 70%, will be negative and we could use other noninvasive alternatives in order to select individuals with polyps for a therapeutic colonoscopy. With this strategy we would perform colonoscopy only in patients with significant lesions and avoid a lot of unnecessary explorations. Otherwise, in our study from 51 subjects included, 18 (35%) had lesions in colonoscopy and only 4 (7%) had significant lesions. CCE identified all of these patients with significant lesions. This could have a positive impact on waiting colonoscopy lists and reduce the care burden of the endoscopy units. On the other hand, less colonoscopy implies less complication. We think that all of these can make the CCE a cost-effective alternative. A great option in CCE will be a same-day CCE and colonoscopy if the patient has polyps, avoiding two colon preparations, but this is logistically difficult for the moment as Adrián-de-Ganzo et al. hypothesize recently [18].

CCE is a well-tolerated exploration and probably its use could increase colorectal cancer screening adherence although this has not yet been demonstrated [23]. Satisfaction level is another key feature in adherence to screening programs. In our study 70% of subjects preferred CCE for screening method. The results of the subjective assessment questionnaire showed that CCE was rated higher than colonoscopy and was perceived as good, very good, or excellent by all patients. In addition, we found that individuals would prefer to repeat CCE rather than colonoscopy for surveillance purposes. Again, we have data about a small number of patients and more studies have to be done.

Colon cleanliness is a crucial aspect related to sensitivity of CCE. Colon preparation usually consists in the administration of a clear liquid diet and a combination of laxatives and prokinetic agents [24]. We used a new low-volume solution consisting of PEG associated with ascorbic acid. This preparation has recently been evaluated with adequate results, achieving excellent or good cleanliness in >80% of patients [25]. In our study, however, overall excellent or good cleanliness was achieved in 60.7% of the patients (70.3% in patients in group 1, MoviPrep booster). Patient tolerance of this preparation was good and the procedure was classified as easier for the participants since a low volume of liquid was ingested. Also we found that PEG + ascorbic boost seems to improve overall colon cleanliness compared to sodium phosphate boost but we have a small size of patients need more studies in order to optimize colon capsule preparation.

More studies are needed to investigate the long-term follow-up of these individuals, as they are at higher risk of CRC and small polyps can be missed. It is unknown whether persons with familial risk might have faster growth and progression of polyps. Only one study has evaluated the cost-effectiveness of CCE in these persons, using a computer model, and concluded that cost-effectiveness depends mainly on the ability of the procedure to improve adherence to CRC screening [24].

The limitation of this study is that it was performed with a first-generation capsule endoscopy. Recently, a second-generation capsule, Pillcam Colon 2, has been developed, which has higher sensitivity and obtains better results in the detection of colonic lesions [21]. The main studies published with first-generation CCE [10, 12, 13] reported sensitivities of 63–88% and specificities of 64–94% in the detection of colonic lesions with high NPVs. Two recent meta-analyses with 626 patients and 837 patients, respectively, found sensitivities for significant polyps of 69–76%, with specificities of 86% and 82% [20, 22]. In our study, CCE detected 4 polyps that were missed on colonoscopy and repeat colonoscopy confirmed only 1 of them as a true positive result. The remainder was confirmed as false positives of CCE. Assessment of polyp size can also lead to confusion. In this respect, the second-generation capsule is an important new advance that allows measurement of polyp size, which will probably decrease the number of false-positive results but not make them disappear completely [26].

In conclusion, CCE is a promising tool that should be considered as an alternative technique in the screening of patients with familial colorectal cancer in order to reduce the number of colonoscopies performed. More studies are needed to understand appropriate screening follow-up intervals and optimize the bowel preparation regimen.

## Figures and Tables

**Table 1:** Study protocol: colon preparation.

	Clear liquid diet

*Day 1*	
12:00 p.m.	200 g carbohydrates + water
17:00 p.m.	200 g carbohydrates + water
19–21:00 p.m.	1 liter PEG + ascorbic + water
*Day 0 *	
7:00 a.m.	1 liter PEG + ascorbic + water
9:00 a.m.	10 mg metoclopramide
9:30 a.m.	*Capsule ingestion*
10:00 a.m.	1° boost- 2/3 l. PEG + ascorbic + water or Sodium Phosphate^*∗*^
13:00 p.m.	2° boost- 1/3 l. PEG + ascorbic + water or sodium phosphate (3 hours after first boost).^Ψ^
	Suppository
17:00 PM p.m.	*Colonoscopy*

^*∗*^(30 minutes after pylorus pass checked by RAPID real-time viewer).

^Ψ^If capsule was not excreted.

**Table 2:** Demographic characteristics of the population.

Sex (M/F), *n* (%)	23 (43.1%)/28 (56.9%)
Age (years), mean (SD)	48.6 (8.9)
NSAIDs or anticoagulant therapy, *n* (%)	7 (13.7%)
Relatives with CRC, mean (SD)	1.42 (0.54)
First degree relative, *n* (%)	33 (64.7%)
Two or more relatives with CRC, *n* (%)	18 (39.6%)
Age at diagnosis of CRC (years), mean (SD)	56.8 (1.8)

**Table 3:** Characteristics of the polyps found in CCE and colonoscopy.

Individuals	Booster	Cleanness in colon segments	CCE polyps	Colonoscopy polyps	Histology
(1)	PEG-Asc	Good	10 mm sigmoid	10 mm sigmoid	Tub-vell

(2)	NaP	Good	8 mm descending colon 3 mm sigmoid	8 mm descending colon 3 mm sigmoid	Tubular adenoma

(3)	NaP	Excellent Good	10 mm cecum 5 mm sigmoid	12 mm cecum 5 mm sigmoid	Tubular adenoma

(4)	PEG.Asc	Fair	8 mm descending colon	7 mm transverse colon	Tubular adenoma
		Fair	5 mm descending colon	5 mm sigmoid	
		Poor	8 mm sigmoid	4 mm rectum	
		Fair	10 mm sigmoid	7 mm recto	
		Fair	4 mm recto	4 mm recto	
			3 mm recto	4 mm recto	

(5)	PEG.Asc	Excellent	3 mm recto	4 mm recto	Tubular adenoma

(6)	PEG Asc	Fair	— 3 mm sigmoid —	5 mm transverse 3 mm recto 3 mm recto	Tubular adenoma

(7)	NaP	Excellent	3 mm sigmoid 3 mm sigmoid 3 mm sigmoid 4 mm sigmoid 2 mm recto 3 mm recto	3 mm sigmoid 3 mm sigmoid 2 mm sigmoid 3 mm recto 3 mm recto 2 mm recto	Hyperplastic

(8)	*NaP*	*Good*	*3 mm recto*	2 mm recto	Hyperplastic
			—	2 mm recto	
			—	2 mm recto	

(9)	*NaP*	Good	6 mm descending	—	Hyperplastic
			5 mm descending	—	
			3 mm recto	3 mm sigmoid	

(10)	*NaP*	Good	2 mm recto	2 mm recto	Hyperplastic

(11)	*NaP*	Good	3 mm descending — — 2 mm recto	2 mm descending 2 mm descending 2 mm descending 2 mm recto	Hyperplastic

(12)	NaP	Good	4 mm sigma	5 mm sigma	Tubular adenoma

(13)	PEG Asc	Poor	5 mm descending 2 mm descending	— —	

(14)	PEG Asc	Good	2 mm transverse 2 mm descending 2 mm descending 7 mm sigmoid	3 mm ascending 3 mm transverse 2 mm sigmoid 5 mm sigmoid	Tubular adenoma Tubular adenoma Hyperplastic Tubular adenoma

(15)	NaP	Fair	5 mm ascending 4 mm descending — —	3 mm ascending 5 mm sigmoid 4 mm sigmoid 2 mm sigmoid	Tubular adenoma

(16)	PEG Asc	Good	—	3 mm sigmoid	Tubular adenoma

(17)	NaP	Poor	— — — —	5 mm transverse 2 mm recto 3 mm recto 2 mm recto	Tubular adenoma Hyperplastic

(18)	NaP	Excellent	— —	3 mm recto 3 mm recto	^*∗*^

(19)	PEG Asc	Good	—	3 mm sigmoid	Hyperplastic

^*∗*^Unrecovered polyps.

**Table 4:** Diagnostic yield of colon capsule endoscopy for detection polyps.

		Prevalence (%)	Sensitivity% (95% IC)	Specificity% (95% IC)	PPV% (95% IC)	NPV% (95% IC)
Polyps	Any size	51 (74%)	66 (61–97)	100	100	89 (79–99)
	≥6 mm	4 (7.8)	100	96 (90–100)	67 (29–100)	100
	≥10 mm	2 (3.9)	100	98 (94–100)	67 (29–100)	100

Adenoma	Any size	12 (23.5)	83 (62–100)	100	100	95 (89–100)
	≥6 mm	4 (7.8)	100	98 (94–100)	67 (29–100)	100
	≥10 mm	2 (3)	100	98 (94–100)	67 (29–100)	100

Advanced adenoma	Any size	2 (3.8)	100	98 (94–100)	67 (29–100)	100
	≥6 mm	2 (3.8)	100	98 (94–100)	67 (29–100)	100
	≥10 mm	2 (3.8)	100	98 (94–100)	67 (29–100)	100

CRC		0				

## References

[1] Butterworth A. S., Higgins J. P. T., Pharoah P. (2006). Relative and absolute risk of colorectal cancer for individuals with a family history: a meta-analysis. *European Journal of Cancer*.

[2] Ng S. C., Lau J. Y. W., Chan F. K. L. (2013). Increased risk of advanced neoplasms among asymptomatic siblings of patients with colorectal cancer. *Gastroenterology*.

[3] Castells A., Marzo M., Bellas B. (2004). Clinical guidelines for the prevention of colorectal cancer. *Gastroenterologia y Hepatologia*.

[4] Piñol V., Andreu M., Castells A., Payá A., Bessa X., Jover R. (2004). Frequency of hereditary non-polyposis colorectal cancer and other colorectal cancer familial forms in Spain: A Multicentre, Prospective, Nationwide Study. *European Journal of Gastroenterology and Hepatology*.

[5] Dove-Edwin I., Sasieni P., Adams J., Thomas H. J. W. (2005). Prevention of colorectal cancer by colonoscopic surveillance in individuals with a family history of colorectal cancer: 16 year, prospective, follow-up study. *British Medical Journal*.

[6] Saraste D., Martling A., Nilsson P. J. (2016). Complications after colonoscopy and surgery in a population-based colorectal cancer screening programme. *Journal of Medical Screening*.

[7] Van Rijn J. C., Reitsma J. B., Stoker J., Bossuyt P. M., Van Deventer S. J., Dekker E. (2006). Polyp miss rate determined by tandem colonoscopy: a systematic review. *American Journal of Gastroenterology*.

[8] Baxter N. N., Goldwasser M. A., Paszat L. F., Saskin R., Urbach D. R., Rabeneck L. (2009). Association of colonoscopy and death from colorectal cancer. *Annals of Internal Medicine*.

[9] Brenner H., Hoffmeister M., Arndt V., Stegmaier C., Altenhofen L., Haug U. (2010). Protection from right-and left-sided colorectal neoplasms after colonoscopy: Population-Based Study. *Journal of the National Cancer Institute*.

[10] Eliakim R., Fireman Z., Gralnek I. M. (2006). Evaluation of the PillCam Colon capsule in the detection of colonic pathology: results of the first multicenter, prospective, comparative study. *Endoscopy*.

[11] Leighton J. A., Rex D. K. (2011). A grading scale to evaluate colon cleansing for the PillCam COLON capsule: a reliability study. *Endoscopy*.

[12] Van Gossum A., Navas M. M., Fernandez-Urien I. (2009). Capsule endoscopy versus colonoscopy for the detection of polyps and cancer. *New England Journal of Medicine*.

[13] Sieg A., Friedrich K., Sieg U. (2009). Is PillCam COLON capsule endoscopy ready for colorectal cancer screening? A prospective feasibility study in a community gastroenterology practice. *American Journal of Gastroenterology*.

[14] Spada C., Riccioni M. E., Hassan C., Petruzziello L., Cesaro P., Costamagna G. (2011). Pillcam colon capsule endoscopy: a prospective, randomized trial comparing two regimens of preparation. *Journal of Clinical Gastroenterology*.

[15] Pilz J. B., Portmann S., Peter S., Beglinger C., Degen L. (2010). Colon capsule endoscopy compared to conventional colonoscopy under routine screening conditions. *BMC Gastroenterology*.

[16] Gay G., Delvaux M., Frederic M., Fassler I. (2010). Could the colonic capsule pillcam colon be clinically useful for selecting patients who deserve a complete colonoscopy? results of clinical comparison with colonoscopy in the perspective of colorectal cancer screening. *American Journal of Gastroenterology*.

[17] Sacher-Huvelin S., Coron E., Gaudric M. (2010). Colon capsule endoscopy vs. colonoscopy in patients at average or increased risk of colorectal cancer. *Alimentary Pharmacology and Therapeutics*.

[18] Adrián-de-Ganzo Z., Alarcón-Fernández O., Ramos L. (2015). Uptake of colon capsule endoscopy vs colonoscopy for screening relatives of patients with colorectal cancer. *Clinical Gastroenterology and Hepatology*.

[19] Lai E. J., Calderwood A. H., Doros G., Fix O. K., Jacobson B. C. (2009). The Boston bowel preparation scale: a valid and reliable instrument for colonoscopy-oriented research. *Gastrointestinal Endoscopy*.

[20] Spada C., Hassan C., Marmo R. (2010). Meta-analysis shows colon capsule endoscopy is effective in detecting colorectal polyps. *Clinical Gastroenterology and Hepatology*.

[21] Eliakim R., Yassin K., Niv Y. (2009). Prospective multicenter performance evaluation of the second-generation colon capsule compared with colonoscopy. *Endoscopy*.

[22] Rokkas T., Papaxoinis K., Triantafyllou K., Ladas S. D. (2010). A meta-analysis evaluating the accuracy of colon capsule endoscopy in detecting colon polyps. *Gastrointestinal Endoscopy*.

[23] Rex D. K., Adler S. N., Aisenberg J. (2015). Accuracy of capsule colonoscopy in detecting colorectal polyps in a screening population. *Gastroenterology*.

[24] Hassan C., Zullo A., Winn S., Morini S. (2008). Cost-effectiveness of capsule endoscopy in screening for colorectal cancer. *Endoscopy*.

[25] Hartmann D., Keuchel M., Philipper M. (2012). A pilot study evaluating a new low-volume colon cleansing procedure for capsule colonoscopy. *Endoscopy*.

[26] Spada C., Hassan C., Barbaro B. (2015). Colon capsule versus CT colonography in patients with incomplete colonoscopy: a prospective, comparative trial. *Gut*.

